# Supplementary material for: Unveiling the Bioactive Potential of the Invasive Jellyfish Phyllorhiza punctata Through Integrative Transcriptomic and Proteomic Analyses
Source: Biomolecules. 2025 Aug 4;15(8):1121. doi: 10.3390/biom15081121 (PMC12383608; doi:10.3390/biom15081121)
Supplement: Supplementary file 1 [file biomolecules-15-01121-s001.zip › Suplementary Material/FileS3/PEPFOLD4/XP_065054274_15176/clusters.html]

 PEP-FOLD clusters


Download archive of all models  here   

|  |  |  |
| --- | --- | --- |
| # | file | sOPEP |
| # cluster 1 - Size: 1 |  |  |
|  | XP\_065054274\_15176-model1 | -32.4353 |
| # cluster 2 - Size: 3 |  |  |
|  | XP\_065054274\_15176-model2 | -32.0166 |
|  | XP\_065054274\_15176-model2.1 | -27.3299 |
|  | XP\_065054274\_15176-model2.2 | -24.5338 |
| # cluster 3 - Size: 1 |  |  |
|  | XP\_065054274\_15176-model3 | -31.85 |
| # cluster 4 - Size: 1 |  |  |
|  | XP\_065054274\_15176-model4 | -30.6367 |
| # cluster 5 - Size: 1 |  |  |
|  | XP\_065054274\_15176-model5 | -27.8798 |
| # cluster 6 - Size: 1 |  |  |
|  | XP\_065054274\_15176-model6 | -27.4707 |
| # cluster 7 - Size: 1 |  |  |
|  | XP\_065054274\_15176-model7 | -27.3893 |
| # cluster 8 - Size: 1 |  |  |
|  | XP\_065054274\_15176-model8 | -26.5041 |
| # cluster 9 - Size: 1 |  |  |
|  | XP\_065054274\_15176-model9 | -26.2829 |
| # cluster 10 - Size: 1 |  |  |
|  | XP\_065054274\_15176-model10 | -26.2121 |
| # cluster 11 - Size: 1 |  |  |
|  | XP\_065054274\_15176-model11 | -26.1187 |
| # cluster 12 - Size: 1 |  |  |
|  | XP\_065054274\_15176-model12 | -25.8725 |
| # cluster 13 - Size: 1 |  |  |
|  | XP\_065054274\_15176-model13 | -25.0586 |
| # cluster 14 - Size: 1 |  |  |
|  | XP\_065054274\_15176-model14 | -24.9802 |
| # cluster 15 - Size: 1 |  |  |
|  | XP\_065054274\_15176-model15 | -24.5722 |
| # cluster 16 - Size: 1 |  |  |
|  | XP\_065054274\_15176-model16 | -24.5571 |
| # cluster 17 - Size: 1 |  |  |
|  | XP\_065054274\_15176-model17 | -24.441 |
| # cluster 18 - Size: 1 |  |  |
|  | XP\_065054274\_15176-model18 | -24.2755 |
| # cluster 19 - Size: 1 |  |  |
|  | XP\_065054274\_15176-model19 | -24.0385 |
| # cluster 20 - Size: 1 |  |  |
|  | XP\_065054274\_15176-model20 | -23.8719 |
| # cluster 21 - Size: 1 |  |  |
|  | XP\_065054274\_15176-model21 | -23.6496 |
| # cluster 22 - Size: 1 |  |  |
|  | XP\_065054274\_15176-model22 | -23.5805 |
| # cluster 23 - Size: 1 |  |  |
|  | XP\_065054274\_15176-model23 | -23.2958 |
| # cluster 24 - Size: 1 |  |  |
|  | XP\_065054274\_15176-model24 | -23.1646 |
| # cluster 25 - Size: 1 |  |  |
|  | XP\_065054274\_15176-model25 | -23.117 |
| # cluster 26 - Size: 1 |  |  |
|  | XP\_065054274\_15176-model26 | -22.7144 |
| # cluster 27 - Size: 1 |  |  |
|  | XP\_065054274\_15176-model27 | -22.6371 |
| # cluster 28 - Size: 1 |  |  |
|  | XP\_065054274\_15176-model28 | -22.5703 |
| # cluster 29 - Size: 1 |  |  |
|  | XP\_065054274\_15176-model29 | -22.5646 |
| # cluster 30 - Size: 2 |  |  |
|  | XP\_065054274\_15176-model30 | -22.4424 |
|  | XP\_065054274\_15176-model30.1 | -20.6256 |
| # cluster 31 - Size: 1 |  |  |
|  | XP\_065054274\_15176-model31 | -22.146 |
| # cluster 32 - Size: 1 |  |  |
|  | XP\_065054274\_15176-model32 | -22.0839 |
| # cluster 33 - Size: 2 |  |  |
|  | XP\_065054274\_15176-model33 | -22.0511 |
|  | XP\_065054274\_15176-model33.1 | -21.2978 |
| # cluster 34 - Size: 1 |  |  |
|  | XP\_065054274\_15176-model34 | -21.5313 |
| # cluster 35 - Size: 1 |  |  |
|  | XP\_065054274\_15176-model35 | -21.3494 |
| # cluster 36 - Size: 1 |  |  |
|  | XP\_065054274\_15176-model36 | -21.2952 |
| # cluster 37 - Size: 1 |  |  |
|  | XP\_065054274\_15176-model37 | -21.2525 |
| # cluster 38 - Size: 1 |  |  |
|  | XP\_065054274\_15176-model38 | -21 |
| # cluster 39 - Size: 1 |  |  |
|  | XP\_065054274\_15176-model39 | -20.8506 |
| # cluster 40 - Size: 1 |  |  |
|  | XP\_065054274\_15176-model40 | -20.723 |
| # cluster 41 - Size: 1 |  |  |
|  | XP\_065054274\_15176-model41 | -20.632 |
| # cluster 42 - Size: 1 |  |  |
|  | XP\_065054274\_15176-model42 | -20.5751 |
| # cluster 43 - Size: 1 |  |  |
|  | XP\_065054274\_15176-model43 | -20.5152 |
| # cluster 44 - Size: 1 |  |  |
|  | XP\_065054274\_15176-model44 | -20.4983 |
| # cluster 45 - Size: 1 |  |  |
|  | XP\_065054274\_15176-model45 | -20.2306 |
| # cluster 46 - Size: 1 |  |  |
|  | XP\_065054274\_15176-model46 | -20.0649 |
| # cluster 47 - Size: 1 |  |  |
|  | XP\_065054274\_15176-model47 | -20.0038 |
| # cluster 48 - Size: 1 |  |  |
|  | XP\_065054274\_15176-model48 | -19.9618 |
| # cluster 49 - Size: 1 |  |  |
|  | XP\_065054274\_15176-model49 | -19.9088 |
| # cluster 50 - Size: 1 |  |  |
|  | XP\_065054274\_15176-model50 | -19.866 |
| # cluster 51 - Size: 1 |  |  |
|  | XP\_065054274\_15176-model51 | -19.7621 |
| # cluster 52 - Size: 1 |  |  |
|  | XP\_065054274\_15176-model52 | -19.6404 |
| # cluster 53 - Size: 1 |  |  |
|  | XP\_065054274\_15176-model53 | -19.1864 |
| # cluster 54 - Size: 1 |  |  |
|  | XP\_065054274\_15176-model54 | -19.1823 |
| # cluster 55 - Size: 2 |  |  |
|  | XP\_065054274\_15176-model55 | -18.9864 |
|  | XP\_065054274\_15176-model55.1 | -12.6545 |
| # cluster 56 - Size: 1 |  |  |
|  | XP\_065054274\_15176-model56 | -18.9707 |
| # cluster 57 - Size: 1 |  |  |
|  | XP\_065054274\_15176-model57 | -18.9625 |
| # cluster 58 - Size: 1 |  |  |
|  | XP\_065054274\_15176-model58 | -18.9494 |
| # cluster 59 - Size: 1 |  |  |
|  | XP\_065054274\_15176-model59 | -18.7077 |
| # cluster 60 - Size: 1 |  |  |
|  | XP\_065054274\_15176-model60 | -18.6536 |
| # cluster 61 - Size: 1 |  |  |
|  | XP\_065054274\_15176-model61 | -18.5002 |
| # cluster 62 - Size: 2 |  |  |
|  | XP\_065054274\_15176-model62 | -18.4261 |
|  | XP\_065054274\_15176-model62.1 | -14.7755 |
| # cluster 63 - Size: 1 |  |  |
|  | XP\_065054274\_15176-model63 | -18.3816 |
| # cluster 64 - Size: 1 |  |  |
|  | XP\_065054274\_15176-model64 | -18.3719 |
| # cluster 65 - Size: 1 |  |  |
|  | XP\_065054274\_15176-model65 | -18.2994 |
| # cluster 66 - Size: 1 |  |  |
|  | XP\_065054274\_15176-model66 | -18.0793 |
| # cluster 67 - Size: 1 |  |  |
|  | XP\_065054274\_15176-model67 | -17.8656 |
| # cluster 68 - Size: 1 |  |  |
|  | XP\_065054274\_15176-model68 | -17.7349 |
| # cluster 69 - Size: 1 |  |  |
|  | XP\_065054274\_15176-model69 | -17.3891 |
| # cluster 70 - Size: 1 |  |  |
|  | XP\_065054274\_15176-model70 | -17.1055 |
| # cluster 71 - Size: 1 |  |  |
|  | XP\_065054274\_15176-model71 | -17.0384 |
| # cluster 72 - Size: 1 |  |  |
|  | XP\_065054274\_15176-model72 | -16.9901 |
| # cluster 73 - Size: 1 |  |  |
|  | XP\_065054274\_15176-model73 | -16.5228 |
| # cluster 74 - Size: 1 |  |  |
|  | XP\_065054274\_15176-model74 | -16.4835 |
| # cluster 75 - Size: 1 |  |  |
|  | XP\_065054274\_15176-model75 | -15.7455 |
| # cluster 76 - Size: 1 |  |  |
|  | XP\_065054274\_15176-model76 | -15.7254 |
| # cluster 77 - Size: 1 |  |  |
|  | XP\_065054274\_15176-model77 | -15.6322 |
| # cluster 78 - Size: 1 |  |  |
|  | XP\_065054274\_15176-model78 | -15.3621 |
| # cluster 79 - Size: 1 |  |  |
|  | XP\_065054274\_15176-model79 | -14.9295 |
| # cluster 80 - Size: 1 |  |  |
|  | XP\_065054274\_15176-model80 | -14.7764 |
| # cluster 81 - Size: 1 |  |  |
|  | XP\_065054274\_15176-model81 | -14.7265 |
| # cluster 82 - Size: 1 |  |  |
|  | XP\_065054274\_15176-model82 | -14.235 |
| # cluster 83 - Size: 1 |  |  |
|  | XP\_065054274\_15176-model83 | -14.2111 |
| # cluster 84 - Size: 1 |  |  |
|  | XP\_065054274\_15176-model84 | -14.1508 |
| # cluster 85 - Size: 1 |  |  |
|  | XP\_065054274\_15176-model85 | -14.067 |
| # cluster 86 - Size: 1 |  |  |
|  | XP\_065054274\_15176-model86 | -13.9648 |
| # cluster 87 - Size: 1 |  |  |
|  | XP\_065054274\_15176-model87 | -13.8362 |
| # cluster 88 - Size: 1 |  |  |
|  | XP\_065054274\_15176-model88 | -13.6848 |
| # cluster 89 - Size: 1 |  |  |
|  | XP\_065054274\_15176-model89 | -13.6171 |
| # cluster 90 - Size: 1 |  |  |
|  | XP\_065054274\_15176-model90 | -13.4703 |
| # cluster 91 - Size: 1 |  |  |
|  | XP\_065054274\_15176-model91 | -13.361 |
| # cluster 92 - Size: 1 |  |  |
|  | XP\_065054274\_15176-model92 | -12.7931 |
| # cluster 93 - Size: 1 |  |  |
|  | XP\_065054274\_15176-model93 | -12.647 |
| # cluster 94 - Size: 1 |  |  |
|  | XP\_065054274\_15176-model94 | -11.9278 |
|  |  |  |
